# Becoming agents for genomic change: genetic counsellors’ views of patient care and implementation influences when genomics is mainstreamed

**DOI:** 10.1038/s41431-024-01686-9

**Published:** 2024-08-29

**Authors:** Trang Thu Do, Melissa Martyn, Belinda McClaren, Alison McEwen, Clara Gaff

**Affiliations:** 1 Murdoch Children’s Research Institute, Melbourne, VIC Australia; 2 Melbourne Genomics Health Alliance, Melbourne, VIC Australia; 3 University of Melbourne, Melbourne, VIC Australia; 4 Graduate School of Health, University of Technology Sydney, Sydney, NSW Australia

**Keywords:** Genetic services, Genetic counselling

## Abstract

Genetic counsellors (GCs) across the world are increasingly transitioning beyond clinical genetics services to meet the growing demands for genomic healthcare. This presents a unique opportunity for GCs to be ‘genomic change agents’ as they work in alternative models of care. Through various innovative models of mainstream care funded through a change program, we explored the views of GCs regarding their position as ‘genomic change agents’ and what may hinder or drive the success of their evolving roles. Guided by the Diffusion of Innovation Theory, we conducted qualitative interviews with all twelve GCs employed by the change program in different models of providing genomics across five specialties in Australia. Audio-recordings of all interviews were transcribed verbatim and analysed using inductive content analysis. Findings show that early in these new roles, participants held varied descriptions of ‘genomics mainstreaming’: some envisioned it as an end state exclusive to medical specialists practicing genomics while others saw the involvement of GCs as crucial. Participants believed they were uniquely positioned to expedite patient access to genomic testing and counselling and enhance medical specialists’ capability to use genomics. Challenges included hesitancy of some medical specialists regarding the value of genomics in healthcare and potential tension arising from distinct perspectives and practice between genetic and non-genetic professionals. Participants anticipated a decline in the standard of care when non-genetic colleagues managed consent discussion and result disclosure. Our study underscores leadership support and peer connection with those in similar roles as essential elements for GCs’ success in mainstream settings.

## Introduction

Genomic technology has rapidly evolved, with genome sequencing increasingly incorporated into healthcare for enhanced prediction of risk, more accurate diagnosis, and more effective treatment for both common and rare conditions [1–3]. Demands for genomic testing and counselling continue to grow, exceeding workforce capacity of clinical genetics services [4]. To meet the rising demands for incorporating genomics into routine care, alternative models of care are needed and will likely involve genetic counsellors working in new ways.

Genetic counsellors (GCs) have transitioned to work outside of clinical genetics services [2]. For instance, in primary care, GCs provide genetic educational outreach and support services to general practitioners [5] or facilitate genetic screening for medically actionable predisposition among rural populations [6]. In cancer care, GCs work alongside oncologists to identify patients who require genetic testing, and counsel ovarian cancer patients [7]. Oncology clinics have formed referral pathways involving GCs leading multidisciplinary review of referrals and acting as a case manager for patients and families throughout the genetic testing process [8]. Further, GCs work in paediatric arrhythmia clinics, screening patients’ medical records, consulting, triaging patients with a primary diagnosis from a cardiologist or neonatologist, and reviewing genomic variants [9]. GCs have also worked in inpatient care across multiple specialties, including general paediatrics, cancer, neonatology, and neurology where they collect samples, discuss genetic testing, provide psychological care, and train resident physicians on test ordering and result interpretation [10, 11].

In these models of care, GCs contribute to wide-ranging positive outcomes for patients and families through improving referral to and patients’ uptake of genetic testing [8], enhancing compliance with guideline-based care [12], reducing patients’ wait time to see a genetic health professional, and hence facilitating and expediting treatment decisions [13, 14]. Through contributions to identifying and triaging patients [9], GCs help reduce unnecessary testing and healthcare costs and increase health-system efficiency [15]. Further, GCs support and educate medical specialists^1^ when it comes to managing complex genomic information, patient education, psychosocial support, or managing patients’ queries and referrals, thereby enhancing case management and patient care [5, 16–18].

While benefits to patients and medical specialists have been reported when GCs are working in these roles, little is known about their experiences. An early study in the United Kingdom (UK) examined the employment of a GC who provided educational outreach to general practitioners [5]. The GC in that model described their experience as both interesting and challenging, as they were anxious about managing a high number of queries in their new role. Another UK-based study [19] offers insights into the benefits GCs gained while working in clinical specialties, for instance, the ability to work autonomously and develop expertise in the specialist areas. However, GCs described challenges, such as limited career progression and feeling isolated. Increased, sometimes unmanageable, workload has also been found to constrain the experience and practice of GCs [20], especially for those working in dual roles [10]. A lack of clinicians’ understanding about the value of genomics in patient care [20] or the nature of GCs’ roles in the specialties [19, 21] have been commonly noted as major difficulties for GCs across the UK and Canada.

Although empirical research on the experiences of GCs working in new settings has emerged, this body of literature is often limited to a single specialty. There is a need to understand the various roles GCs can play in delivering genomic change and generate evidence for establishing and sustaining alternative models of integrating genomics into medical care. Drawing upon the first phase of a qualitative study, in this paper we explore the views and experiences of GCs working as ‘genomic change agents’ to support the adoption of genomics in a single health system in Australia. These GCs were employed by a change program which set up innovative models of care in neurology, nephrology, haematology, transplant, and cardiology.

## Methods

### Theoretical framework

Our study is informed by Rogers’ Diffusion of Innovations (DOI) theory [22] and the model of diffusing innovations in healthcare proposed by Greenhalgh et al. [23]. These models suggest some innovations spread more easily than others depending on the innovation’s attributes, including its relative advantage, complexity, compatibility, observability, and trialability [22, 23]. The DOI theory acknowledges the key role of a change agent in influencing the uptake of a new practice [22]. Change agents serve as a communication link between a change agency or innovation developers and target users [22], by forging interpersonal relationships with potential adopters, and through social interactions, influencing the adopters’ behaviours about the innovation and the dissemination process [23]. Despite the prominence of the DOI in implementation research, there is less attention paid to conceptualising and describing the change agents’ role in influencing the adoption of a novel intervention [24, 25]. Focusing on GCs as change agents, we aim to explore their perspectives of new models of delivering genomic testing and counselling in medical specialties (the *innovation*) and their new role in the process of introducing change into health services.

### Australian healthcare context

The healthcare system of Australia comprises public and private sectors whereby citizens and permanent residents are covered within the public health system through Medicare, which is funded by the federal government [26]. People can access genetic services via the public system free-of-charge if the genetic testing is considered medically appropriate [27]. GCs in Australia mainly work in publicly funded clinical genetics departments, but new positions have emerged for GCs in non-genetic specialties and private clinics [26]. The minimum requirement to practice as a GC in Australia is the completion of a two-year Master’s Degree in genetic counseling (or previously a one-year Graduate Diploma program) [27]. Upon completing the degree, an individual is eligible to work as an Associate Genetic Counsellor and can become a Certified Genetic Counsellor after completing certification [28].

### Procedures

With ethics approval granted by Royal Melbourne Hospital’s Human Research Ethics Committee (Reference Number: HREC/80793/MH-2021) and the University of Melbourne’s Office of Research Ethics and Integrity (Reference number: 2023-26642-40071-3), a research team member contacted potential participants to inform them about the study and invite them for an interview. Participants could choose to be interviewed online (via Zoom), via phone, or in person. Prior to interview, participants were sent a participant information sheet and consent form which they signed and returned before any interviews. All interviews were conducted by the first author who is not a GC and had not known any of the participants prior to the interviews.

Informed by the DOI theory and based on our literature review and clinical and research experience, we developed an interview schedule (included in the Supplementary file) with open-ended questions and prompts to guide the conversations. As suggested by the existing literature, there is a lack of a unified working definition of genomics mainstreaming and related guidelines, which may lead GCs to feel unsupported in the specialties and render them in positions where other colleagues may have inappropriate requests for their responsibilities [19, 20]. We therefore asked participants to provide their understanding of mainstreaming genomics and how they conceptualised their role in the specialties. We then inquired about the barriers and enablers participants could anticipate or had experienced working in those positions.

### Participants

**Fig. 1:**
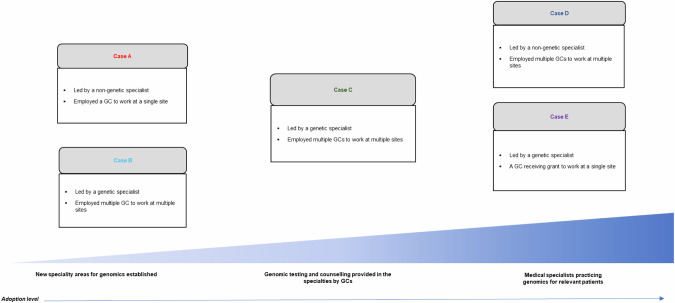
New models of genomic care: providing genomic testing and counselling in five medical specialties.

All twelve GCs eligible for the study due to their funded mainstream role were recruited to participate in our interviews. Cases have been used to describe characteristics of the new models of care at a single site or across multiple sites at different health services. These were initiated and led by either a genetic professional (a geneticist or a GC) or a medical specialist, and aimed to achieve various levels of genomics adoption (Fig. 1). One participant each was recruited from Case A, Case B, and Case E; three from Case C; and six from Case D.

The recruited GCs were affiliated with five clinical genetics services in an Australian metropolitan city where they continued to work while being seconded to a medical specialty for a 12- or 18-month period in a part-time role. The participants’ scope of practice in the specialist clinics varied: some participants continued to see patients, while others were more likely to focus on upskilling medical specialists about genomics (for example, through advising about testing, providing education, participating in multidisciplinary team meetings, or managing a support line). Some participants were also involved in research-related tasks in their new role. The recruited sample included ten women and two men who possessed a diverse range of working experience (Table 1).

**Table 1:** Participant characteristics.

	Number of participants (total *n* = 12)
Years of experience working as a GC	
Less than 5 years	3
5 to 9 years	5
10 years and above	4
Certified or Associate	
Certified	8
Associate	4
Primary affiliation	
Public genetics department	11
Private service	1
Number of days/week working in the specialty	
1.5 day or less	4
2 to 2.5 days	5
3 days	3

### Data analysis

Each interview, 30-to-50-min duration, was recorded and transcribed verbatim. Deidentified transcripts were uploaded into NVIVO 14.0 for data management and analysed using inductive content analysis [29]. The first author read all the transcribed data and coded segments of the interviews to identify broader categories related to the research aims. These chunks of data were later coded line-by-line and refined into subcategories. A subset of three transcripts were independently coded by a co-author. Throughout this process, the whole team met regularly to compare how we had interpreted the data using the DOI theory and discussed any coding differences until we reached consensus about a code structure. The first author then applied this structure to re-examine all interview transcripts and identified the final categories and subcategories, and the relationships between them.

## Findings

### Understanding of genomics mainstreaming and the new models

Participants described the different factors that motivated them to work in the specialty, ranging from known evidence from existing models in oncology to potential opportunities for the genetic counselling profession (Table 2). Our interviews suggest wide variations in the ways the participants conceptualised and identified the various actors involved in mainstreaming genomics although most participants envisioned mainstreaming as an end state where genomics was used in the specialties. Some GCs characterised mainstreaming by using words that suggested the autonomy and independence of medical specialists in practicing genomics:

[I]n the purest sense of mainstreaming, you shouldn’t really be relying on a genetic counsellor, they should be available absolutely, but really mainstreaming in its purest sense is non-genetic specialists is doing everything independently as much as possible. (#07)

**Table 2:** The motivations to work in a new role in the specialties.

Motivating factor	Illustrative quotes
Previous knowledge or experience in the specialty area	I always may have had a really strong interest in *specialty* genetics. I really enjoy as a subspecialty of genetics. I think it’s really interesting and previously quite underserved area of genetics. (#06) I was sometimes going to the *specialist* outpatient clinic. I was seeing patients there and talking about their genetic test results or consenting them to the project. (#01)
Contributions to a new area for genomics	It does feel different to just being in a role that exists and you’re just one of the people who’s doing the job. Whereas in the *specialty* I feel like we’re creating something… which is pretty cool. (#11)
Acquisition of new knowledge	[P]harmacogenomic testing is probably something that isn’t even happening in clinical genetics… because it didn’t exist in genetics very much beforehand. We’re all learning about pharmacogenomics together. (#12)
Preparation for prospective changes of the genetic counselling profession	[M]ainstreaming of genetic testing is kind of the future of the profession of genetic counselling, and it sounds like a really exciting opportunity to be part of the team, part of the project that was implementing the mainstreaming of genetic testing. (#03)
A solution to address fast-growing demands for genomic testing	[O]ur genetic service itself is extremely stretched for resources. We have more referrals than we can possibly manage. And so, this idea of mainstream genetic testing is really one possible solution. We’re very keen to explore, to try and help manage the growing needs for genetic services. (#06)
Inspiration from the success of mainstreaming genomics in oncology	[I]t’s a really worthwhile service. We provide that sort of rapid testing. For patients I think, it is really useful. And there’s really good data [from cancer mainstreaming] showing that it’s beneficial to patients with a lot of other treatment plans going on… And so I think we’re really excited to be part of something new. (#08)

Likewise, another participant refuted the notion of having a GC work at a specialist clinic as a mainstream component:

When I think about mainstreaming, I think about non-genetic health professionals [doing] testing and consenting patients for testing. I don’t see that what I’ll be doing necessarily be mainstreaming because there will be a genetic professional in the room. (#01)

In contrast, other participating GCs deviated from the definition that solely emphasised the role of medical specialists to one that considered the setting where genomic testing was offered:

[M]ainstreaming to me is more externalising the genetic testing outside of genetics (services) itself while maintaining [GCs] being there to support the specialists along the way, so they still feel well supported… particularly for the complicated ones. (#06)

Not only speaking about GCs’ “behind-the-scene” or “in the background” roles in genomics mainstreaming, some went to recognise the central role of GCs in that practice:

I know that they’ve been talking about the embedded model [GCs seeing patients in a specialist clinic]. So of course, that is a form of mainstreaming where you have a genetic counsellor involved in a clinic outside of genetics (services). (#02)

In those participants’ opinions, they adopted a more inclusive definition of mainstreaming by describing it as a collaborative approach involving multiple actors, including both genetic and non-genetic specialists, in the practice of genomics in the specialties.

### The perceived value of working in the specialties

Despite variations in the ways participants defined their role and conceptualised ‘mainstreaming,’ they all recognised the significance of their new role on patient outcomes in terms of expediting patient access to genomic testing and counselling and expanding the utility of genomics for treatment and care (Table 3). Specifically, they mentioned a significant decrease of wait time for patients, since patients were able to consult a GC as part of their specialist care or having genomic testing managed by their treating physicians rather than being referred to an external department. A participant noted:

I anticipate patients won’t have to wait for more than one or two weeks [as compared to a six-to-nine month wait if referred to a genetics service] to be seen. I’d like to be able to say that I can see you next week or even this week. (#10)

**Table 3:** Perceived value of GCs’ roles in the specialties.

Value of GCs’ roles	Illustrative quotes
*Impacts on patients*	
Expedite patients’ access to genomic testing and address the wait time challenge for patients	[F]irstly either the patient has to ask, or their doctor has to mention it, which does not always happen. I would expect that in my experience, anyway, it’s quite rare for the *specialists*, to facilitate the genetic testing themselves… then get referred to a genetics clinic… and that appointment might be on average, 5 to 9 months away. So there’s a bit of time for the disease to progress. (#02) [T]he idea of mainstreaming where you’re already seeing a *specialist* when you have *an organ* disease, and then they’re able to and doing the testing if needed or referred to.” (#09)
Ensure continuity of care	And then you’re obviously getting that continuity of care by that *specialist* that has seen that patient. And then you’re seeing that same patient. And they’re able to do that genetic testing.” (#09)
Alter routine care practice for patients	[H]istorically, the genetics wasn’t even part of the *specialty* at all… People were diagnosed with clinical diagnoses and descriptive diagnoses based on the way their *organ* looks under an ultrasound or under from a biopsy… [A] fact of mainstreaming meaning far more patients can have access to that as part of their care has the huge potential to alter their care. (#04)
Improve patients’ level of comfort and care experiences overall	I also think that bringing us into the room, as I said, like this will build rapport with the patients. And then, if rather than seeing me separately or a geneticist separately, being invited into the room it seems to be a better model where it seems to be or like multi-disciplinary room… Obviously that’s like better communication. (#03)
Modulate patients’ misconceptions about genetics departments and the utility of genomics	There’s often a lot of misconception that clinical genetics we’re a research [department], that we’re just interested in finding, doing genetic discoveries and doing studies on families rather than understanding of using genetic testing as a tool for informing medical management… Because we [genetics services] are external, sometimes they don’t always appreciate the motivations behind why they’ve been referred. (#06)
*Impacts on medical specialists*	
Improve medical specialists’ confidence and capability in integrating genomics into patient care	[We are] kind of being there to support the specialists along the way, so they still feel well supported, and feel that they’re able to ask questions and know that we are still here, particularly for the complicated ones, although people just need more support in general. So they don’t kind of feel like they need to take it all on themselves. (#06)
Building up the autonomy of medical specialists so that they could use genomics with minimal GCs’ support	So I will be sort of there and available, as and when required, and probably quite a lot in the beginning. But then, over time, I plan to hopefully fade away into the background as they become more confident and competent to do it themselves. (#07)

Participants also saw the potential of their role in replicating the success of mainstreaming models in oncology regarding setting up a new standard of care:

[T]here’s certainly other areas like *certain medical*^2^ conditions where perhaps you could streamline this process for patients from certain clinics to just really kind of keep our wait list not piling up… like you see in mainstreaming in cancer where so much of our genetic testing is now done through mainstreaming. (#02)

In addition to the benefits for patients, participants articulated the potential impacts on medical specialists due to their regular interaction with those colleagues. Participating GCs described how they were well-positioned to establish active cross-specialty communication: “[I]t will kind of encourage more open dialogue between genetic services and the clinicians involved in these clinics.” (#02)

For some, working in the specialties was viewed as an opportunity to model the genetic counselling skillset to medical specialists:

[T]he relational sort of skills that genetic counsellors have and so, that’s one thing that I hope will come out as we move from just ordering test to returning results and working with families once there’s something to talk about. That will be supported and acknowledged as a critical part of the process. (#04)

Those GCs believed that they were ideally placed in a position to demonstrate the relational aspects of care that genetic professionals often take pride in. This could be an effective strategy to increase the observability of a practice among the medical specialists they collaborated with when GCs were physically present in the specialties.

### Barriers and enablers to GCs’ new speciality roles

#### Barriers

Study participants identified multiple factors that may or had already impacted upon the success of their positions in the specialties (Table 4). Among those challenges, the hesitancy of some medical specialists about the utility of genomics in healthcare was often noted as a major barrier, which may interfere with their engagement with the GCs and ultimately influence the motivation for change:

I think it’ll just be interesting to see how the team works, and how open and accepting they are to having a kind of genetic counsellor be involved. And what their attitudes are about genetic testing… You could say that there might be some people who think it’s no point. (#02)

**Table 4:** Challenges and enablers to GCs’ new roles in the specialties.

Influences	Illustrative quotes
*Challenges*	
Medical specialists’ hesitancy in engaging with the project	[S]ome of our *specialist* colleagues are feeling a bit resistant to this project, and that they feel like they might not have the capacity to include this as part of their usual clinical workload, which is, I think, understandable. I think everyone in public health they [are] overworked… (#05)
Lacking the interaction to ascertain the level of genomics-related knowledge of medical specialists	Some of the specialists just aren’t reaching out that much, and so I’m not sure if it’s because they don’t think their patients would benefit from genetic testing, and it’s not useful for them, so they’re not asking. Or if it’s because they [do] not feel confident, or they might have some concerns, but they don’t feel comfortable bringing it up. I don’t know. (#05)
Differences in professional views and practice between the genetic and medical specialists	[A] huge gap between how a genetic health professional would interpret a result and explain that result to a patient, and then use that result with a family compared to a specialist’s interpretation and understanding of a result. (#04)
Tension for clinician education due to the differences between the two professions	I think education about result, interpretation, and how to use it in your clinical practice is going to be a challenge because I think that there’s nuance that can be missed, and if you miss it, you might never know that you’ve missed it because you just accept the thing that’s evolved or as the result. (#04)
The feeling of isolation	I’m a little silo. I feel like I’m a little bit separate from the *specialty* and genetics, waving the flag for *specialty* genetics on my own and I think that’s not gonna go away, because even when I’m in the *specialist* clinics regularly, I’m the only genetics person there.” (#07)
*Enablers*	
Inter-professional collaboration	I think that I would like them to learn what a genetic counsellor actually does and the value of genetic counsellors in a service like this so they could appreciate the skillset we bring and the level of support we can give. (#10)
A clear scope of roles for both GC and medical specialists involved in mainstreaming	[I]t’s really important that they also recognize this separate role that would remain for the genetics clinic what’s appropriate for mainstreaming, and when to refer on and that I wouldn’t expect a *specialist* to offer predictive testing to unaffected family members. (#05) [E]veryone knows that you are there to work together… not be a gatekeeper for genetic testing… but more there to enrich the experience with family and the patient. (#01)
Medical specialists showing momentum in orienting the delivery of a new model of care	It might be that the clinicians need to determine when the best timing is for an appointment. And hopefully, that’s something that can be done and not just relying on my availability kind of thing. (#02)
Leadership endorsement conducive to forging the connection between a genetic counsellor and a team of medial specialists	[T]he head [of a clinic] has sort of attended those [introductory meetings], and reiterated their belief that this [mainstreaming model] is important, which I think has been really integral to the [specialists] who don’t know who I am. Most of them they don’t know who I am, but their head feels this is important, so hopefully [that] helps keep them on side, keep them interested. (#07)
Easy access to the project leaders and supervisors in the specialist clinic	[T]o be able to speak with the geneticists and *specialists* about complex cases is really important. [Name of the project lead] is running the project across many different sites. And so, making sure these people I can go to… [H]aving people I can contact with complex cases, both complex genetics and medically, but also complex psychosocially as well, is really important. (#08)
Peer connection	I think implementation isn’t something that genetic counsellors naturally think about… So they’re really having to draw on different skills. And so I think it’s nice to have a community you can come to and talk about that sort of stuff. (#12) I want to connect to the genetic counsellors that are on this project to create a chat thing as well, so that there is that connection between the counsellors to support each other, and also to discuss anything that works and doesn’t work so that we can then take that away to our site. (#09)

Notably, in many interviews participants pointed to the differences in professional views and practice between the genetic and non-genetic specialists, which would potentially cause tension for GCs’ roles and affect patient care. For instance, many spoke about the genetic professionals’ emphasis of viewing patients as a family unit rather than an individual and their greater attention to counselling or patients’ psychosocial aspects. But participants did not regard these as the priorities for many medical specialists, as one GC articulated this differentiated view:

[T]he sort of challenges that come up are the different ways that genetic counsellors are trained compared to doctors in terms of the emphasis that is placed on counseling and appointments, so the importance of picking up the cues that the patients are giving and asking them questions about how they’re feeling and trying to delve into getting to know them a bit more broadly than just organising the testing. (#011)

Likewise, another participant spoke from their previous observation in an oncology clinic and raised a concern over the loss of certain care aspects for patients when genomic testing was managed by medical specialists:

Without the support of genetics service, it doesn’t identify the family members that may miss out on screening, and not tested for cancer even if they got a high-risk mutation, or even might not have been tested for the right gene. (#08)

Tension might also arise due to the different views between GCs and the project leads about the fit of the innovation within the local context when a single model of care was implemented across multiple sites. Project leads, as described here, designed and led the projects within the funded change program. This tension was noted as a potential challenge for sustainability:

We want to create something that’s going to be sustainable. And there’s a bit of a discord there. So what you just find is that the project [leaders] says: “This is how we’re going to do the thing.” And then at the local site, that’s not really going to work here… They [the leads] have some sort of defined ideas about how you might deliver genetics in these particular areas of medicine. (#12)

Further, participants mentioned multiple logistical barriers they had encountered that may compromise the success of their new role in the specialties. These include, for example, laborious administrative tasks related to enacting a grant or a lack of designated workspace in the specialist clinics. Notably, some participants reported the challenge when juggling in the dual role between the genetics service and the specialty:

[E]ven though I’m supposed to be working on this [mainstream] project three of the days and then genetics two of the days, inevitably you just get approached by everybody all the time about my normal role [in the genetics department] … I was finding being in the office every day, I think it was hard for my colleagues and hard for me to actually protect my time. (#07)

#### Enablers

Participants considered their own efforts and preparation as vital to address those aforementioned barriers and succeed in their specialty role. One GC spoke about an intuitive strategy that could be helpful when working as a new addition to a specialist team:

[I]t’s very important to kind of speak up when you can and also shut up when you should. It’s not my job to bulldoze in there and change practice, but to really kind of ask and query, and say: “Oh, well, did you consider this?” So, gentle challenging from both ends. (#02)

Clarity around the scope of work was also helpful to form reasonable expectations from medical specialists about the role that GCs undertake in specialist settings, as that participant continued: “I think it’ll be important to ensure that they are aware that we are not testing facilitators, we’re decision-making facilitators.” (#02)

Importantly, GCs also saw the role of medical specialists in orienting the model delivery, which would allow GCs’ support to be compatible with those colleagues’ needs, thereby encouraging receptivity to change:

I will ask them what they want in terms of the feedback, whether they want a phone call with results, or do they want just a letter given that I’m kind of an internal person in their team for the duration of the study. How do they want that to be different to a referral to an external service? (#10)

Further, most participants mentioned the role of leadership support and commitment was integral to create a welcoming environment for GCs and endorse the relevance of genomics for patient care (Table 4). Participants spoke of the collective ownership they needed from the project leads who designed and led the innovative models funded by the change program in which they were employed. The desired ownership was described in terms of the ability to contribute to the design and delivery of the respective model:

I think it’s supporting, hearing those issues and advocating for you to have a role in how the program’s set up. So, having some responsibility in the way that mainstream program is rolled out. (#08)

Crucially, in many participants’ opinions, peer connection constituted a source of support for GCs to overcome the challenges encountered in the specialties. Specifically, a participant spoke about the benefits of building a network with other GCs who worked in similar roles:

[F]eeling that I am still connected, even though these people don’t even work [in the] same service and are doing different things, but just not feeling that sense of I’m the only one pioneering something. It’s not just me. It’s others as well. (#07)

The importance of connecting with peer GCs who have similar mainstream experience via a community was believed to both provide emotional support and benefit participants’ learning and professional growth (as quoted in Table 4). Those enabling factors along with the barriers identified earlier should be considered for efforts seeking to support GCs working beyond the confines of clinical genetics services.

## Discussion

This paper examines the perspectives of GCs working in medical specialist clinics to facilitate the use of genomics and the multitude of influences on their new specialty roles. Our interviews suggested participants held varied, sometimes contrasting definitions of ‘genomics mainstreaming’ practice: some perceived it as a practice exclusively involving medical specialists while others recognised the crucial involvement of genetic counsellors. This lack of a common understanding might create difficulty for GCs to work towards a shared vision of an end goal. It may also have implications for GCs’ communication with medical specialists and get them engaged in the innovation. Further, we also note that participants often shaped their understanding of mainstreaming based on their prior experience or observation of models implemented in cancer care. This frequent reference is reflective of the dominance of the evidence base generated from genetics-mainstreaming innovations in oncology where oncologists were held responsible for offering and consenting patients for genetic testing with minimal involvement of a clinical genetics team [13, 20, 30, 31]. It suggests there are limited mainstream models for GCs to draw upon when moving into another specialty area. Considering this, we identify a critical need for funding alternative genetic counselling models and support for publications from those cases to enrich the evidence on the effectiveness of genomics mainstreaming in other specialties.

Two main and interlinked barriers were frequently mentioned as impeding the success of GCs’ roles in specialist settings. Most notably, participants spoke about the hesitancy of medical specialists when it comes to incorporating genomics in patient care. This is a common theme in the existing literature which often found that medical specialists were not convinced about the relevance of genomics for their practice or medical care [20]. Meanwhile, some participants experienced a lack of engagement from medical specialists, which might be challenging for GCs to foster their interactions needed for communicating about the relative advantage and demonstrating the benefits of genomics in order to persuade an uptake of a new practice [22, 32]. The situation might be explained by the clinicians’ limited understanding of GCs’ presence in the specialty when the relationship had not yet been established [21]. However, we expect this would be overcome as the collaboration progresses.

An important step in supporting integration of genomics in specialist care is negotiating what good genomic care ‘looks like’ in the specialities. This process of ‘re-negotiation’ of genomics ‘fits’ it to the new practice setting and enhances the likelihood of sustainment [22]. Participants anticipated tensions arising as genomics was used in the specialities due to the different perspectives and practices of the different professions (ie. genetic counsellors and medical specialists). Genetic counsellors expressed concerns over a decline in the standard of care, insufficient counselling for patients, or a lack of attention to psychosocial needs of family members when medical specialists conducted consent discussion and communicate genomic test results with patients. Resolving tensions is a critical part of the innovation process [22, 33]. As genomics is increasingly delivered outside of clinical genetic services, how can GCs as change agents demonstrate and transfer the skillsets pertaining to their profession while remaining receptive to the ‘reinvention’ among medical specialists who incorporate genomics in a way that align with their established practices and local resources? We will explore this question and prompt others to do so to inform strategies that encourage and sustain active uptake of genomics in medical specialties.

For GCs to act as ‘change agents’ they need to integrate successfully into the medical specialty clinics they are working in. Our interviews identify multiple factors that support successful integration. Participants mentioned the vital role of clinic leaders in creating a welcoming environment for GCs and endorsing the value of genomics mainstreaming, which resonates with previous studies emphasising senior management as enablers to successful implementation of health innovations [34, 35]. In addition, participants stressed the necessity of connecting with other genetic colleagues with experience working beyond the confines of clinical genetics. In their opinions, peer connection served two important purposes: emotional and professional. Emotionally, building a network with colleagues in similar positions provided GCs with the contacts with whom they could relate to and helped them overcome the feeling of isolation while working in the specialty as a minority voice [19]. Such connection was also perceived as professionally important as GCs could share the hurdles encountered in their roles, develop best practices, and seek advice for improvement from other GCs working across different specialist settings. Guided by those findings, we have established a community of practice for GCs employed to support the adoption of genomics in diverse specialty areas. We expect this would become an effective forum for peer advice, collaborative learning, and competence building that helps GCs succeed in a rapidly changing profession.

Our study has some limitations. GCs participating in our interviews worked in new roles created as part of funded projects. While these positions were established in a structured and innovative way, the participants recruited in our study might not represent the wider group of GCs employed to work in the specialties through the typical funding pathways. However, the strength of this research is the inclusion of participants from different models and our findings hold the implications for setting up alternative models of delivering genomic testing and counselling across hospital sites and specialties.

## Conclusion

Our paper expands the conceptual understanding of GCs’ evolving roles beyond clinical genetics services. As our participants suggested, the new role of GCs as agents diffusing the use of genomics in specialist care has not yet been well-defined. This may limit discussions around forming a new professional identity for GCs while they take on employment opportunities in response to growing demands for genomic healthcare. We hope our findings can inform implementation research and practice that attempts to identify support and competencies needed to transition GCs into new working areas. Successful implementation might also benefit from exploring the (mis)alignment in the perspectives towards the innovation, but also the relational dynamics among different actors (i.e innovators, change agents, and prospective adopters) involved in the process of introducing change, which we recommend for future research to focus on.

## Supplementary information

Interview Guide

## Data Availability

The dataset generated during the overarching study is not yet complete, because data collection is still ongoing. However, the data analysed for this paper could be made available from the corresponding author on reasonable request.

## Footnotes

1 In this paper we use “medical specialist” to refer to medically trained healthcare professionals without a genetic background.

2 We replace the name of a specialty area with a general term (left in italicised text in quotes) to protect the anonymity of participants or the clinic they were employed by.

## References

[1] Middleton A, Marks P, Bruce A, Protheroe-Davies LK, King C, Claber O, et al. The role of genetic counsellors in genomic healthcare in the United Kingdom: a statement by the Association of Genetic Nurses and Counsellors. Eur J Hum Genet. 2017;25:659–61. 28327572 10.1038/ejhg.2017.28 PMC5518913

[2] Ormondroyd E, Mackley MP, Blair E, Craft J, Knight JC, Taylor J, et al. Insights from early experience of a Rare Disease Genomic Medicine Multidisciplinary Team: a qualitative study. Eur J Hum Genet. 2017;25:680–6. 28327571 10.1038/ejhg.2017.37 PMC5427178

[3] White S, Jacobs C, Phillips J. Mainstreaming genetics and genomics: a systematic review of the barriers and facilitators for nurses and physicians in secondary and tertiary care. Genet Med. 2020;22:1149–55. 32313152 10.1038/s41436-020-0785-6

[4] Hoskovec JM, Bennett R, Carey M, DaVanzo J, Dougherty M, Hahn S, et al. Projecting the supply and demand for certified genetic counselors: a workforce study. J Genet Counseling. 2018;27:16–20. 10.1007/s10897-017-0158-8 29052810

[5] Drury N, Bethea J, Guilbert P, Qureshi N. Genetics support to primary care practitioners—a demonstration project. J Genet Counseling. 2007;16:583–91. 10.1007/s10897-007-9096-1 17497110

[6] Christensen KD, Bell M, Zawatsky CL, Galbraith LN, Green RC, Hutchinson AM, et al. Precision population medicine in primary care: the Sanford Chip experience. Front Genet. 2021;12:626845. 33777099 10.3389/fgene.2021.626845 PMC7994529

[7] Rana HQ, Kipnis L, Hehir K, Cronin A, Jaung T, Stokes SM, et al. Embedding a genetic counselor into oncology clinics improves testing rates and timeliness for women with ovarian cancer. Gynecologic Oncol. 2021;160:457–63. 10.1016/j.ygyno.2020.11.003 33229043

[8] Kentwell M, Dow E, Antill Y, Wrede CD, McNally O, Higgs E, et al. Mainstreaming cancer genetics: a model integrating germline BRCA testing into routine ovarian cancer clinics. Gynecologic Oncol. 2017;145:130–6. 10.1016/j.ygyno.2017.01.030 28162234

[9] Helm BM, Freeze SL, Spoonamore KG, Ware SM, Ayers MD, Kean AC. The genetic counselor in the pediatric arrhythmia clinic: Review and assessment of services. J Genet Counseling. 2018;27:558–64. 10.1007/s10897-017-0169-5 29079892

[10] Clark CR, Reyes K, Ormond KE, Caleshu C, Moscarello T. US Genetic counselors’ perceptions of inpatient genetic counseling: A valuable model for medically complex patients. J Genet Counseling. 2021;30:1683–94. 10.1002/jgc4.1435 34124811

[11] Magness E, Magoulas P, Moscarello T, Ma D, Helm BM, Mizerik E. Characterization of genetic counselor practices in inpatient care settings. J Genet Counseling. 2021;30:1181–90. 10.1002/jgc4.1401 33713511

[12] Senter L, O’Malley DM, Backes FJ, Copeland LJ, Fowler JM, Salani R, et al. Genetic consultation embedded in a gynecologic oncology clinic improves compliance with guideline-based care. Gynecologic Oncol. 2017;147:110–4. 10.1016/j.ygyno.2017.07.141 28800943

[13] Pederson HJ, Hussain N, Noss R, Yanda C, O’Rourke C, Eng C, et al. Impact of an embedded genetic counselor on breast cancer treatment. Breast cancer Res Treat. 2018;169:43–6. 29349711 10.1007/s10549-017-4643-4

[14] Yanes T, Sullivan A, Barbaro P, Brion K, Hollway G, Peake J, et al. Evaluation and pilot testing of a multidisciplinary model of care to mainstream genomic testing for paediatric inborn errors of immunity. Eur J Hum Genet. 2023:31:1125–32. 10.1038/s41431-023-01321-z PMC10545723 36864115

[15] Wakefield E, Keller H, Mianzo H, Nagaraj CB, Tawde S, Ulm E. Reduction of health care costs and improved appropriateness of incoming test orders: the impact of genetic counselor review in an academic genetic testing laboratory. J Genet Counseling. 2018;27:1067–73. 10.1007/s10897-018-0226-8 29427196

[16] Emery J, Hayflick S. The challenge of integrating genetic medicine into primary care. BMJ. 2001;322:1027–30. 11325768 10.1136/bmj.322.7293.1027 PMC1120183

[17] Battista R, Blancquaert I, Laberge A-M, Van Schendel N, Leduc N. Genetics in health care: an overview of current and emerging models. Public Health Genomics. 2012;15:34–45. 21734357 10.1159/000328846

[18] Patch C, Middleton A. Genetic counselling in the era of genomic medicine. Br Med Bull. 2018;126:27–36. 29617718 10.1093/bmb/ldy008 PMC5998955

[19] Quinn E, Mazur K. The experiences of UK-based genetic counsellors working in mainstream settings. Eur J Hum Genet. 2022;30:1283–7. 35918538 10.1038/s41431-022-01158-y PMC9343813

[20] Hallowell N, Wright S, Stirling D, Gourley C, Young O, Porteous M. Moving into the mainstream: healthcare professionals’ views of implementing treatment focussed genetic testing in breast cancer care. Fam Cancer. 2019;18:293–301. 30689103 10.1007/s10689-019-00122-y PMC6560008

[21] Slomp C, Morris E. GenCOUNSEL Study, Price M, Elliott AM, Austin J. The stepwise process of integrating a genetic counsellor into primary care. Eur J Hum Genet. 2022;30:772–81. 35095102 10.1038/s41431-022-01040-x PMC8801315

[22] Rogers E. Diffusion of Innovations. 5th ed. New York: Free Press; 2003.

[23] Greenhalgh T, Robert G, Macfarlane F, Bate P, Kyriakidou O. Diffusion of innovations in service organizations: systematic review and recommendations. milbank Q. 2004;82:581–629. 15595944 10.1111/j.0887-378X.2004.00325.x PMC2690184

[24] Leathers SJ, Spielfogel JE, Blakey J, Christian E, Atkins MS. The Effect of a Change Agent on Use of Evidence-Based Mental Health Practices. Adm Policy Ment Health Ment Health Serv Res. 2016;43:768–82. 10.1007/s10488-015-0694-1 PMC4838563 26487393

[25] McCormack B, Rycroft-Malone J, DeCorby K, Hutchinson AM, Bucknall T, Kent B, et al. A realist review of interventions and strategies to promote evidence-informed healthcare: a focus on change agency. Implement Sci. 2013;8:107. 24010732 10.1186/1748-5908-8-107 PMC3848622

[26] Dwarte T, Barlow‐Stewart K, O’Shea R, Dinger ME, Terrill B. Role and practice evolution for genetic counseling in the genomic era: The experience of Australian and UK genetics practitioners. J Genet Counseling. 2019;28:378–87. 10.1002/jgc4.1053 30629777

[27] Ormond KE, Laurino MY, Barlow‐Stewart K, Wessels TM, Macaulay S, Austin J, et al. Genetic counseling globally: Where are we now? Am J Med Genet Part C. 2018;78:98–107. 10.1002/ajmg.c.31607 PMC5947883 29575600

[28] (HGSA) HGSoA. Genetic Counselling Training and Accreditation. 2023. https://www.hgsa.org.au/Web/Web/ET/Genetic-Counselling.aspx?hkey=975b23ce-fa8e-485a-a7ee-fc88aab9a60d.

[29] Hsieh H-F, Shannon SE. Three approaches to qualitative content analysis. Qualitative Health Res. 2005;15:1277–88. 10.1177/1049732305276687 16204405

[30] O’Shea R, Rankin NM, Kentwell M, Gleeson M, Salmon L, Tucker KM, et al. How can Australia integrate routine genetic sequencing in oncology: a qualitative study through an implementation science lens. Genet Med. 2020;22:1507–16. 32461668 10.1038/s41436-020-0838-x

[31] George A, Riddell D, Seal S, Talukdar S, Mahamdallie S, Ruark E, et al. Implementing rapid, robust, cost-effective, patient-centred, routine genetic testing in ovarian cancer patients. Sci Rep. 2016;6:29506. 27406733 10.1038/srep29506 PMC4942815

[32] Martyn M, McClaren B, Janinski M, Lynch E, Cunningham F, Gaff C. It’s something I’ve committed to longer term”: The impact of an immersion program for physicians on adoption of genomic medicine. Patient Educ Counseling. 2021;104:480–8. 10.1016/j.pec.2020.10.013 33268232

[33] Haring M, Freigang F, Amelung V, Gersch M. What can healthcare systems learn from looking at tensions in innovation processes? A systematic literature review. BMC Health Serv Res. 2022;22:1–20. 36307839 10.1186/s12913-022-08626-7 PMC9617372

[34] Best S, Brown H, Lunke S, Patel C, Pinner J, Barnett CP, et al. Learning from scaling up ultra-rapid genomic testing for critically ill children to a national level. npj Genom Med. 2021;6:5. 33510162 10.1038/s41525-020-00168-3 PMC7843635

[35] Taylor N, Clay-Williams R, Hogden E, Braithwaite J, Groene O. High performing hospitals: a qualitative systematic review of associated factors and practical strategies for improvement. BMC Health Serv Res. 2015;15:1–22. 26104760 10.1186/s12913-015-0879-z PMC4478709

