# Supplementary material for: Becoming agents for genomic change: genetic counsellors’ views of patient care and implementation influences when genomics is mainstreamed
Source: Eur J Hum Genet. 2024 Aug 29;32(12):1606–14. doi: 10.1038/s41431-024-01686-9 (PMC11606944; doi:10.1038/s41431-024-01686-9)
Supplement: Supplementary file 1 — Interview Guide [file 41431_2024_1686_MOESM1_ESM.docx]

Genetic Counsellor Interview Guide

**Introduction**

Thank you for making the time to participate in this interview with Melbourne Genomics. This interview aims to explore the changing roles of Genetic Counsellors, in particular in facilitating the wider use of genomics in clinical care. We also want to know what support has been/will be useful in helping you in these roles, as well as any potential challenges or barriers you have experienced/may experience when working outside of a clinical genetics service.

Before we start this interview, may I confirm you have read and returned the consent form. Do you have any questions? May I ask if you are happy to progress with the interview today?

I am now going to start recording. Please note that we will only use the audio recording. If it’s easier for you to speak with a camera off, please kindly do so.

**[*** START recording]**

May I start by asking you some demographic questions:

- What is your highest level of education?
- Where do you mainly work? Which hospital? Which department?
- What is your job title at this main role?
- How long have you been working in your current role? How long have you been working as a genetic counsellor?

***Background***

1. Can you tell me a bit about your current or most recent position in the genetics service/department? What are your main tasks?
2. The term mainstreaming is being used a lot at the moment, what’s your understanding of ‘mainstreaming’?
3. Have you worked in a role outside the genetics service? Would you describe it as a mainstreaming role?
4. Can you tell me more about that role? How did you know about that role? What attracted you to apply for this role?
5. How many days will you work in that role? Where (which service/clinic/clinical specialty) will you be based during those days?
6. What did/do you do in this role?

***Perception of the new GC model (the ‘innovation’)***

1. Thinking about the specialty you have worked with, what do you know about usual pathways for patients to access genetics?
2. What do you understand are the current challenges with accessing genetics for patients at the moment?
3. How does/will your role (at/with the clinical specialties) contribute to addressing those challenges?
4. How will your role contribute to supporting the adoption of genomics in that speciality?

***Implementation***

1. What do you expect to/have you achieved in this role?
2. As compared to your current/previous roles in the genetics service/department, what changes have you experienced in terms of the way you practice?
3. What changes have your seen for the patients? For the hospital/clinic?
4. We’re interested to know more about your interaction with the non-genetics health professionals you work with. How do you work with them in your new role?

*Prompts:*

- What works well? What isn’t working well? /What has helped overcome this barrier/issue?

1. How does your interaction with non-genetics HCPs impact their use of genomics (e.g attitudes, confidence, skills, and expertise)?
2. Do you have any challenges or difficulties in your new role?
3. What have you done to prepare for this role?
4. In your new role, when you have a complex case, who do you go to seek professional advice/support? Do you have a supervisor in genetics? How do you work with them?
5. How about a supervisor in the speciality: do you have one?
6. How do you maintain the connection with your colleagues in the genetics service/department?
7. What practical or professional support do you need to successfully work in this new role?
8. What needs to be done/support is needed to sustain use of genomics outside clinical genetic services?

***Closing***

Is there any other subject or topic you think we need to discuss to better understand your new role?

Do you know any other genetic counsellors who we might get in touch with and invite to participate in our interview for this research study?
